# Reliability Assessment Approach for the Quality of Railroad Subgrade

**DOI:** 10.3390/ma15051864

**Published:** 2022-03-02

**Authors:** Janusz Vitalis Kozubal, Piotr Wyborski, Matylda Tankiewicz, Igor Gisterek

**Affiliations:** 1 Faculty of Civil Engineering, Wroclaw University of Science and Technology, 50-370 Wrocław, Poland; janusz.kozubal@pwr.edu.pl (J.V.K.); igor.gisterek@pwr.edu.pl (I.G.); 2 Department of Civil Engineering, Wrocław University of Environmental and Life Sciences, 50-375 Wrocław, Poland; matylda.tankiewicz@upwr.edu.pl

**Keywords:** subgrade, static load test, deformation modulus, reliability

## Abstract

The static load plate test is the standard subgrade acceptance test for new or modernised railway lines. Examinations are performed at regular spacings on the track section and a lack of acceptance for even a single test disqualifies a section, forcing remedial treatments on the whole section. In this paper, a nondeterministic description of stiffness related to the spatial characteristics of acceptance measurement results is proposed for a more rational assessment of substructure quality. The concept is based on geostatistical analysis and one-dimensional distributions of stiffness values. The paper also proposes a new concept of rail infrastructure acceptance based on a reliability index already codified in European standards. The functioning of the postulated criterion is presented on the example of an existing railway line and the actual test results.

## 1. Introduction

The railway subgrade is an important part of the construction of a railway line. Depending on the course of the route and terrain, the tracks are found directly on original subsoil or on anthropogenic soil structure. In the case of old routes, the problems of preserving the homogeneity and quality of the substructure are particularly significant [1,2]. In addition, the increasing speed of trains leads to higher expectations for all components of the track [3,4]. Adapting existing railways to changing standards and guidelines, especially in terms of achievable speeds and axle loads, is now a significant issue. In many cases, track bed investigations are necessary, not only in preparation for the construction, reconstruction and modernisation of a railway line, but also in the case of failure [5]. Damage to the substructure occurs during the operation of a route for various reasons, such as changes in soil and water conditions, design errors, execution errors and construction limitations.

Soil as a track bed is characterised by high variability, including variability in time, which is related to the influence of external factors such as climate conditions or exposure to dynamic effects of rail traffic. Under certain circumstances, it is possible for there to be not only a deterioration but also an increase in the bearing capacity of the subgrade after a certain period of use. This is caused by the compaction of the soil layers as a result of dynamic influences from passing trains. Therefore, for practical confirmation of the theoretical assumptions of newly designed structures and improvement of old ones, it is necessary to build testing sections on test tracks or on sections of active railway lines, where theoretical parameters are subject to final verification. A similar procedure applies to the design of innovative improvement structures and subgrade drainage. In the case of existing routes, in order to properly assess the causes of damage to the track structure and substructure, it is necessary to carry out load-bearing capacity tests on the substructure, which are invasive in relation to the track superstructure.

Due to the reasons mentioned above, the primary approach used in subgrade design is empirical investigations, mainly in situ. The geotechnical testing methods used in railway construction do not differ from those used for other geotechnical structures or road infrastructures. However, it is not clearly stated whether such a procedure is fully justified, not least because of the different ways of transferring forces and loads from trains to the ground [6]. In addition, due to the local specifics (rolling stock, speeds and construction technology), national guidelines have been developed for testing the subsoil of railway lines. In Poland, basic works include the textbooks by A. Wasiutyński [7] and K. Wątorek [8] and works by J. Nowkuński [9], J. Sysak [10] and E. Skrzyński [11]. The common recommendations of the UIC (International Union of Railways) and the associated European standards of the EN series were only developed in the 1960s and 1970s. Important works discussing the topic of subgrade include—but are not limited to—books by Popp [12], Indraratna et al. [13], Li et al. [14] and Correia et al. [15]. Regarding Polish textbooks, conditioned to the national standard, the most relevant ones are Skrzyński [11] and Grulkowski et al. [16].

In the international practice of physical testing of the substructure, the static plate load test is accepted as the basic test [6]. Widely known problems and difficulties connected with static plate testing, significant costs of testing and the long time needed to carry it out properly mean that there is a continuous search for a method that will allow the replacement of it with tests carried out by other methods, which will give results with an acceptable level of probability. The commonly used methods of monitoring the substructure and quality of earthworks are based on a scheme where a sufficient number of tests confirming the quality of the work are carried out by means of measurable parameters. This makes it possible to classify the quality of the work as satisfactory or in need of improvement, i.e., by increasing compaction or by adding admixtures or replacing soil with material, guaranteeing the achievement of the assumed mechanical properties.

The aim of the work is to propose an approach that will make it possible to reduce costly, time-consuming and cumbersome field investigations while adequately assessing the bearing capacity of the subgrade. The paper presents a reliability-based approach. The methodology of reliability estimation has been intensively developed in geotechnical tasks [17,18,19,20] for a significant period of time and is successfully applied in geotechnical design. It is also supported by recent normative acts [21,22]. The key similarity of the presented approach is the reliance on probability theory and the classical definition of the failure region boundary. The mathematical apparatus is also common. The difference is important and crucial and corresponds with the existing railroad standards. It combines the qualitative analysis of the track bed with the safety system, and the assumed limiting vulnerabilities are a generalised description of the track bed condition. This way, the method is called reliability based on the quality parameter, in contrast to load-limit-based reliability.

Investigations of the horizontal spatial variability of the deformation modulus using geostatistical methods have been successfully performed in the past [23,24]. However, the application of these methods to the study of railway or road substructures is a novelty; publications on this issue have been published only recently [25,26]. An unquestionable innovation resulting from this article is a proposal to calculate the reliability with the use of geostatistical methods in issues related to the railway subgrade. The proposed method is based on standard subgrade field tests but takes into account spatial geostatistical relations in the ground. Therefore, it is also possible to predict the state of the substructure beyond the test points. It is based on the correlation between the values of the subgrade modulus as a function of their mutual distance and the generated random values of the modulus in the dense grid. The geostatistical analysis of the obtained values based on reliability assumptions allows for conclusions on the subgrade quality in the serviceability limit-state context in a more extensive way than based purely on test results. The paper includes results of the subgrade quality assessment for a selected railway route section (from West Pomerania, Poland) using the proposed technique with the determination of the required scope of subgrade improvement for the assumed criteria. The issue of selecting the method of substrate improvement based on the results obtained, due to the multiplicity of techniques used and the complexity of the issue, was not considered.

## 2. Materials and Methods

### 2.1. Rail Subgrade Testing

**Figure 1:**
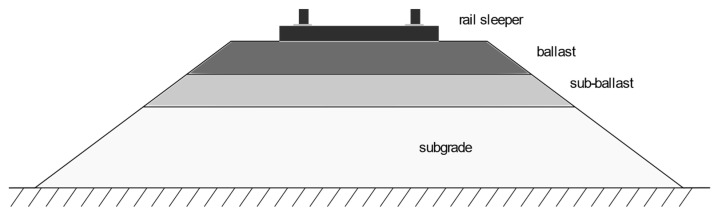
Construction of the railway substructure.

The purpose of the substructure (subgrade) is to transfer loads without permanent deformation to the subsoil from passing rail vehicles, the weight of the rail itself and the layers above it. The subgrade usually consists of natural soil and a thin layer of additional soil material, which is required to provide the planned track path. A typical embankment substructure is shown in Figure 1, where the superstructure layers, i.e., ballast and subballast, are placed on top of the original soil, on which the railway road is placed.

Due to different construction techniques and route paths, the track subgrade may contain different types of soil. Typically, it is constructed of aggregates < 31.5 mm when low water permeability is expected, and of coarser fractions, e.g., 4–31.5 mm, when drainage is required. Such materials provide sufficient bearing capacity and are most suitable to support the ballast layer and ensure required drainage.

As mentioned in the introduction, subgrade quality examinations are carried out in various situations, both on newly built and existing lines. The basic test to evaluate the quality of the subgrade of railroads is the static plate load test. The test is performed by loading the ground in the field with a circular steel plate and allows evaluation of the deformability and the load capacity of the soil. The settlement of the plate is measured by a tester consisting of a carrier frame with a sensing arm and dial gauge. As a counterbalance, a heavy vehicle is used. For each loading step, the corresponding settlement of the plate is recorded. From the load-settlement graph, the primary and secondary deformation modulus (*E_v_*_1_ and *E_v_*_2_) are determined. The test characterises the zone to a depth of 0.30–0.50 m below the plate and it is commonly used for roads and railways. The detailed procedure is described, e.g., in [27]. Depending on the country (region) there are different regulations for subgrade investigations. The work is based on European and local standards [28,29]. Excerpts from these regulations are included in the appendices to [30], which is the mandatory document for national railways in Poland. It describes a static load test with a 300 mm plate, and the number of control points per track length is given. According to this approach, the deformation index *I*_0_ is calculated from the measured values of the moduli *E_v_*_1_ and *E_v_*_2_, and the quality assessment is performed on the basis of the index *I*_0_ and the modulus *E_v_*_2_. In this study, it was decided to base the quality of subgradeonly on the values measured directly in the tests, i.e., both strain moduli.

### 2.2. Variogram Estimators

In the case of linear constructions such as railways, ground investigations are carried out at regular intervals, which is time-consuming and expensive. As a result, the values of the deformation modulus are known only at selected points. In this approach, the results of the study provide an incomplete picture of the changes in the values of the deformation modulus in the railroad axis. To obtain a description of the variation in soil modulus between these points without additional testing, a geostatistical approach can be used. This is possible if the distribution of values is assumed to be an ergodic stationary process. The classical geostatistical approach imposes random fields on the whole longitudinal profile in such a way that the generated values in the profile are autocorrelated with the empirically obtained values. The overlapped random fields in the profile can be described in general by:

(1) $$z\left(s\right)=\mu \left(s\right)+e\left(s\right),$$

where *μ*(*s*) ≡ *E*[*z*(*s*)] is a mean function that is continuous and defined and *e*(*s*) is a random error with zero mean and satisfies the stationarity assumption. A frequently used stationarity hypothesis is weak stationarity, which can be represented as follows:

(2) $$C\left(s_{i}-s_{j}\right)=\mathrm{cov}\left[e\left(s_{i}\right),e\left(s_{j}\right)\right],\;$$

where *C* is the covariance function. It can be concluded that the covariance between *z*-values at any two locations depends only on their mutual position. Another important assumption is intrinsic stationarity. Variograms used to describe it are as follows:

(3) $$2\gamma \left(s_{i}-s_{j}\right)=\mathrm{var}\left[e\left(s_{i}\right),e\left(s_{j}\right)\right],$$

where 2*γ* denotes the variogram function. The variogram represents the dependence ratio of a feature as a function of distance in the normalised Euclidean space *‖h‖* for isotropic phenomena or as a function of distance and direction, assuming anisotropy for phenomena in two and more dimensions. The variogram estimator can be described as:

(4) $$2\hat{\gamma }\left(h\right)=\frac{1}{N\left(h\right)}\underset{N\left(h\right)}{\sum }{\left(z\left(s_{i}\right)-z\left(s_{j}\right)\right)}^{2},$$

where the formula *N*(*h*) denotes the number of all pairs (*z*(*s_i_*) *− z*(*s_j_*))^2^ that are distanced by *‖h‖*. For practical reasons, semivariograms, which are defined as half of the variogram *γ*(*h*), are quite often used [31]. It is a measure of nonsimilarity between points observed at a given location *z*(*s_i_*) and *z*(*s_j_*), as opposed to covariance, which describes similarity. The semivariogram provides information about the spatial continuity and variability of the random function.

In the subgrade quality assessment problem, semivariograms were used to determine the autocorrelation along the rail line created by the testing points. The soil parameters determined at these points were used to create an empirical semivariogram, using the least-squares method and the Gauss–Newton algorithm as a nonlinear fitting method. With these tools, issues related to the influence of local extremes on the results can be avoided. The next step is the selection of a suitable theoretical semivariogram for an accurate spatial prediction of the ground parameters. Cases where the dependence model has a defined semivariogram are relatively rare.

The presented procedure is a standard approach used for several reasons [32], such as to provide a conditional negative specification for a semivariogram, which is necessary for the variance of the prediction error to be non-negative at every point in the space [33]. The most important factor in the selection of the semivariogram model should be convergence to the empirical semivariogram. This can be verified by the reliability function or the least-squares method (LSM). In some cases, other factors such as model flexibility or computational simplicity may be taken into account. The model can be selected from an extensive library of models. The basic parameters of many of the theoretical semivariogram models used are range *r*—the distance over which the resulting values are flattened; and sill *s*—the value the semivariogram reaches beyond distance *r*.

According to theoretical models, if the distance between two points is close to zero then the semivariogram value should be zero. However, sometimes, as the separation distance decreases, the semivariogram values do not approach zero. This phenomenon is called the nugget effect and describes the variability between samples at very small distances [34]. Whether the phenomenon occurs depends on the measurement error or the spatial variability of the ground at distances smaller than the sampling interval, or both simultaneously. The magnitude of the nugget effect consists of two components: the geological nugget effect (GNE) and the sampling nugget effect (SNE). The most commonly used theoretical semivariogram models and the nugget effect are shown in Table 1.

In this paper, in order to obtain probable values of stiffness in the railway track axis, a model of spatial variability described by a semivariogram, being a non-negative function and zero mean value, was used. The set of data obtained in this way is a realisation of a one-dimensional random field. Its values are conditioned by points of known stiffness. For the generation of the field, the algorithm of sequential simulation of a Gaussian conditional field in the Euclidean space for an assumed ergodic and isotropic process was applied. In the discussed issue, the generated points were uniformly distributed on the considered line. The sequential algorithm formulated in this manner is very efficient and works correctly for cases in a large scale. The method uses only data and values simulated from the local neighbourhood to approximate the conditional distribution. In this work we have only proposed a certain set of functions representing the relationships most commonly observed in nature and engineering. In situations with more diverse substrate, other functions would be more appropriate. The proposed scheme thus emphasises the method rather than its implementation, avoiding overly rigid rules that limit potential applications.

### 2.3. Probability of Failure

A random process is a function in a probabilistic space of random ***X*** variables. When this set consists of time-dependent realizations, then it is a stochastic process. In this article, a random event is considered as a stationary function *F*(***X***) with values defined as the set of states of the process. Process states should be understood as defined: *F*(***X***) < 0 failure or not fulfilling acceptance criteria; *F*(***X***) = 0 a limit state; and *F*(***X***) > 0 functioning or fulfilling acceptance criteria. An object, treated as a primary concept in a probabilistic process, can be assigned to:

(a) a category of simple structural elements or structure (e.g., pile, column, retaining wall, anchoring items, elementary subgrade section);
(b) a category of complex objects consisting of simple objects related by mechanical or geometric features.

The probability of failure of a simple object *p_f_* is defined as *p_f_* = *P*(*F*(***X***) ≤ 0), whereas reliability is a property of an object that states whether it works correctly (fulfills all assigned functions and actions) under specific service conditions. Probability in most cases has a small value, so it is more convenient to use a measure of the reliability index *I_β_*. It is defined with respect to probability by the following relationship [21]:

(5) $$p_{f}=\Phi_{0}\left(-I_{\beta }\right),$$

where *Φ*_0_ is the cumulative distribution function for a standard normal probability distribution (with mean 0 and standard deviation 1). According to European guidelines [22], the reliability index required for a structure depends both on the expected costs of protection and the consequences of a potential failure (Table 2).

Complex objects have a definite structure only if it is possible to determine the reliability of the elements and their dependencies. There are two basic types of such structures: serial and parallel. If a system failure occurs when all its components are damaged, then such a structure is called a parallel. The serial structure means that each failure of the system’s components (e.g., simple objects or their sets) is the cause of failure of the whole system. In the presented method this model was used in the basic analysis. If the acceptance criteria are not met for any of the distinguished sections of the subgrade, no acceptance is given for the entire segment under investigation. A threshold variant of the serial system is also presented, where the existence of a number of adjacent elements that do not meet the acceptance criteria together was assumed as a condition for system failure.

To perform reliability considerations, it is necessary to define characteristics such as the potential renewability or reparability of an object. In this paper, only repairable objects were dealt with. Hence, it was possible to build an iterative procedure, and the concept of failure also included nonfulfillment of acceptance criteria. In the strict sense, failure of infrastructure components means a permanent loss of functional or mechanical properties. The processes analysed were also treated as strictly stationary, meaning that their values were not dependent on the position of the reference point on the time axis.

Depending on the available statistical information about the process under study, there are many methods for determining the probability of failure. The methods functioning in design and proposals for future regulation are well-described and classified [35]. Methods can be divided into four levels:

- Level 0—deterministic;
- Level 1—partially probabilistic methods, statistical description of the object by determining safety factors as coefficients modifying the values of loads and capacities;
- Level 2—approximative methods, estimation of the probability of failure by means of safety factors determined from analytical relationships;
- Level 3—fully probabilistic methods, determination of safety factor based on numerical simulations.

The paper focuses on the application of a fully probabilistic approach to determine the probability values of not meeting the required quality criteria. These techniques include the use of a known probability density function of failure, response method methodology (RSM) [36,37,38,39,40], first- and second-order reliability methods (FORM/SORM) [41] and Monte Carlo methods [42,43]. Further, the considerations are based on the crude Monte Carlo method.

Directly assessing the probability of failure is extremely difficult. Many variables are involved, hence direct construction of a CDF with an imposed boundary condition is problematic, especially in the presented case of a track substructure, where each of the analysed points along the length of the studied section is a random variable and the adopted reliability system is based on the Bayesian concept. We have not applied FORM-type methods in this work due to the uncertainty associated with the transformation of random variable distributions to the standardised space. The ambiguity is due to its nature; it may depend on the ordering of the variables in the random data vector. The consequence of this may be different forms of the boundary surface which are affected by generating different values of failure probability. FORM/SORM methods give good results when there is only one computational point and the boundary function is of class C1/C2 and not strongly nonlinear. The Crude Monte Carlo (CMC) method was used in presented work as the numerical integration to find the solution; despite the high dimensionality of the task, this approach is robust to the unusual shape of the limit function, and is also applicable when its form is unknown in the probability hyperspace. For the points and for the entire cross section, the number of exceedances of the failure condition is examined. This allows us to determine the probability of failure to meet the adopted objective criterion for substructure quality.

### 2.4. Proposed Scale of Substructure Quality Assessment

The study by Baumgartner [44] was used as a starting point to assign the consequences of damage to a railway route. This compilation of both infrastructure and rolling stock costs, despite many years since publication, is still often adopted as a reference. This is due to its detailed cost assessment for elements covering all aspects of the railway network (rails, trains, tunnels, bridges, stations and maintenance of these elements) for a large area (EU and USA). Table 3 summarises the estimated costs for constructing a complete railway line. Such compilations are important for the railway industry and are often used for cost estimation [45,46,47].

Another concept of assigning damage consequences other than cost is one in which the purpose of the route—its category—is the main quantifier. For the purpose of the work, the classification of conventional railway lines used in Poland and related substructure elements has been adopted from [48,49]:

- Trunk lines (K0)—traffic volume of over 25 million Mg/year, passenger-train speeds of <200 km/h and goods-train speeds of <120 km/h;
- Primary lines (K1)—traffic volume of 10–25 million Mg/year, passenger-train speeds of <120 km/h and goods-train speeds of <80 km/h;
- Secondary lines (K2)—traffic volume of 3–10 million Mg/year, passenger-train speeds of <80 km/h and goods-train speeds of <60 km/h;
- Lines of local importance (K3)—trainload of up to 3 million Mg/year, passenger-train speeds of <60 km/h and goods-train speeds of <50 km/h.

This classification is the basis for technical guidelines for designing and constructing railway infrastructure facilities.

It was decided to use a combination of the two criteria presented above: cost according to Baumgartner’s scale and categorisation of lines in relation to the reliability index values from Table 2. The reliability index values were assigned to the railway line categories on the basis of an evaluation of the consequences of failure as a supply-chain disruption corresponding only to economic damage. The methodology was based on a matrix of averaged performance costs assigned to the adopted classification of railway roads. In order to determine the reliability coefficient, a cost vector was used, using a linear scaling of the costs associated with topographical difficulties to the required reliability index. After some corrections to match the results to the European standards, the classification presented in Table 4 was obtained. This is a simplified model, which should be treated as a proposal.

**Figure 2:**
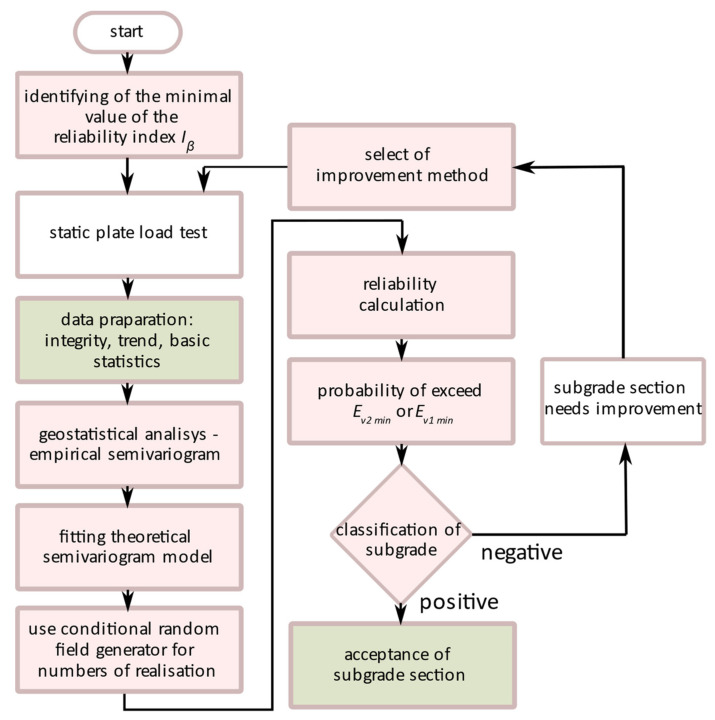
Flow chart of the concept of the subgrade acceptance procedure.

A method of implementation of these very general reliability suggestions in the design practice is shown by the algorithm in Figure 2. After determining the section of the substructure to be assessed and identifying of the class of the section according to the adopted classification, the minimal value of the reliability index beta (Table 4) for the substructure is obtained. The iterative procedure starts with the first in situ tests of the substructure performed with a static plate load test. In the next step, geostatistical analysis is carried out to obtain theoretical semivariograms for the elastic properties of subsoil in the section. Using the procedure described previously, a conditional random field is repeatedly generated for points spaced at a certain distance from each other, corresponding to the distance between the railway axes. It is described by a determined geostatistical relation. European or national standards allow the adoption of an objective criterion to disqualify a test point. In the proposed procedure, the minimum value of *E_v_*_1_ or *E_v_*_2_ is taken as a criterion. For the points and for the whole section, the number of exceedances of the failure condition is tested, e.g., by the Crude Monte Carlo (CMC) method. It allows for the determination of the probability of not fulfilling the adopted objective criterion of the substructure quality. If the reliability index for a point or a section is higher than expected, it means that the execution is correct and further track works can be carried out. Otherwise, improvement works should be carried out in the area where the objective criterion is not met with a given probability. Once the additional tests confirm the quality of the modified subgrade are completed, the calculation procedure shall be repeated. The whole process is continued until approval is obtained at all points specified.

## 3. Application of the Methodology—Case Study

### 3.1. Investigated Section and Test Results

The railway route section located in West Pomerania (Poland) was the subject of research and analysis. The field test covered a section of 9100 m in length and was carried out prior to the planned modernisation works. The investigated object was selected for improvement due to its poor technical condition and the planned upgrade of the railway line (from K1 to K0). Most of the route runs on an embankment except for a 2200–2800 m section which is located on a level surface as a low embankment. The route is free of horizontal and vertical curves and terrain obstacles; the whole section has a gradient of less than 1‰ and is located in an area with a homogeneous geological structure. The subsoil was found to be composed of various types of soils characteristic to the North European Plain (Polish Plain) and the embankment structure was made of sandy soil. Such a section was chosen in order to limit the impact of terrain variability and its effect on the results obtained.

**Figure 3:**
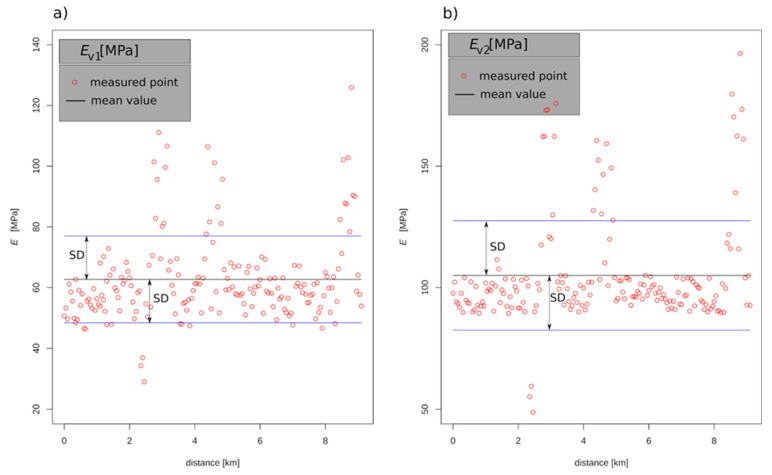
Results of the static load plate test on the selected section: (**a**) *E_v_*_1_; (**b**) *E_v_*_2_.

**Figure 4:**
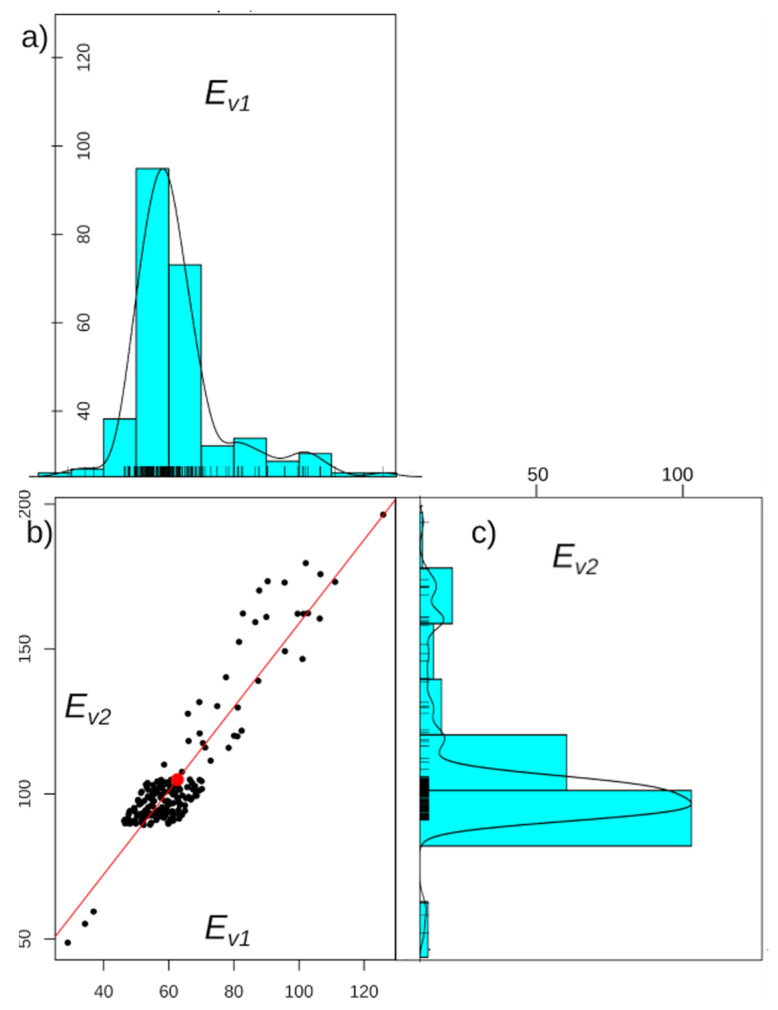
Results of the static load plate test on the selected section: (**a**) histogram of modulus *E_v_*_1_ values; (**b**) values of *E_v_*_2_ versus *E_v_*_1_; (**c**) histogram of modulus *E_v_*_2_ values.

A series of static plate load tests were performed on the investigated section according to the Polish guidelines [30]. A total of 183 tests were carried out at 50 m spacing. The results are shown in Figure 3 and Table 5. The values of the *E_v_*_1_ modulus range from 28.99 to 125.90 MPa with a mean value of 62.68 MPa and a standard deviation of 14.29 MPa. The values of the *E_v_*_2_ modulus range from 48.70 to 196.50 MPa with a mean value of 104.99 MPa and a standard deviation of 22.53 MPa. As can be observed in Figure 3, the vast majority of results are within the ±1 standard deviation range. In order to better illustrate the results obtained, Figure 4 presents histograms of the values of the modules *E_v_*_1_ and *E_v_*_2_ (Figure 3a,c) and their correlation (Figure 3b). In this configuration, the linear correlation between the parameters can be seen, as well as points of particular concern with small values of the moduli. The red point in Figure 4b is the mean value and the red line is a line fitted by the least-squares method. The concentration of points in one group (Figure 4b) results from a strong mutual correlation of the measurements as understood by Pearson. The closer to the line *E_v_*_1_ = *E_v_*_2_, the greater the degree of correlation. The correlation between *E_v_*_1_ and *E_v_*_2_ values is significant and equal to 0.92. When the variability of a parameter is high, it is suggested to separate sections which can be approximated by a linear trend.

### 3.2. Semivariograms and Probability of Failure

**Figure 5:**
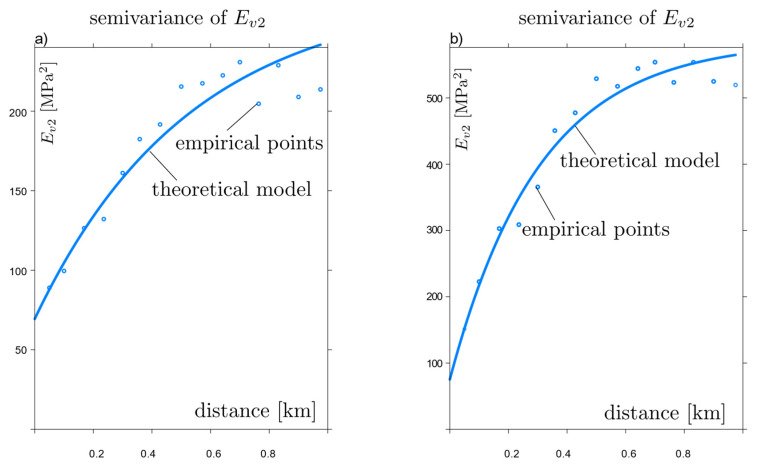
Theoretical and empirical semivariograms: (**a**) for *E_v_*_1_, (**b**) for *E_v_*_2_.

Based on the results of the field study, empirical and theoretical semivariograms were established according to the procedure described in Section 2.2. Figure 5a,b show semivariograms of stiffness values measured in situ with fitted theoretical models. In the two cases studied, an exponential model [50] from Table 1 combined with a nugget effect was used to describe the variability. The results are presented in Table 6. The geostatistical models reproduce a powered exponential covariance structure with a significant randomness of the measured values, as evidenced by the value of high nugget effects (14–33%).

**Figure 6:**
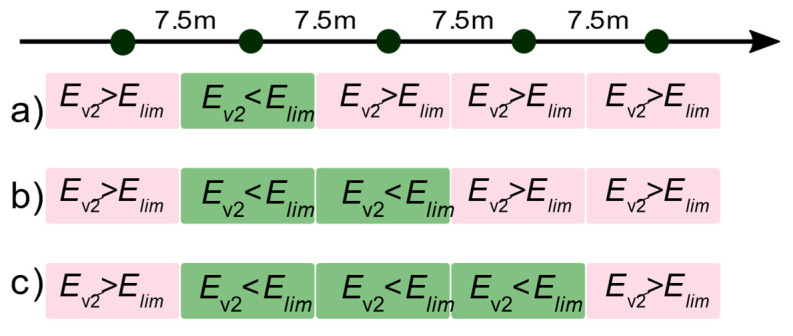
Diagram of reliability test points (7.5 m sections) and possible variants of non-achievement of criterion: (**a**) single point—7.5 m—Mode(7.5); (**b**) two adjacent points—15.0 m—Mode(15), (**c**) three adjacent points—22.5 m—Mode(22.5).

The rail track analyses adopt a load spacing per track at 7.5 m intervals. The examined subgrade was divided into sections, which gives 1215 test points along the track axis. It was assumed to be a serial reliability system, i.e., the existence of a single point or a number of adjacent elements that do not meet the acceptance criteria (expressed in stiffness of the subgrade) is treated as a system failure. The value of *E_v_*_2_ was used as an objective criterion. The technical adequacy criterion of the section is expressed by the condition:

(6) $${\left\{E_{v2}\right\}}_{i}\geq E_{lim},$$

where *i* is a number from 1 to 1215 describing the experimental values of modulus the *E_v_*_2_ for the 7.5 m sections. Figure 6a provides a schematic overview of the test points with possible options for not meeting the objective criterion. In the case when at two (Figure 6b), three (Figure 6c) or more subsequent points the condition Equation (6) is not fulfilled, the mechanical condition of the track–structure–substructure system poses a higher risk of stability. These cases, labelled as Mode(7.5), Mode(15) and Mode(22.5) and so on, can be treated as independent events in the reliability system sense.

According to the established theoretical semivariogram for the investigated points of the subgrade (*E_v_*_2_), describing the variability of the phenomenon, draws of possible values of *E_v_*_2_ between the points were performed, maintaining the values measured in the field. The obtained set of drawn and measured values is denoted as {*E′_v_*_2_} and the failure condition Equation (6) can now be represented as:

(7) $${\left\{E_{v2}^{\prime }\right\}}_{i}\geq E_{lim}.$$

**Figure 7:**
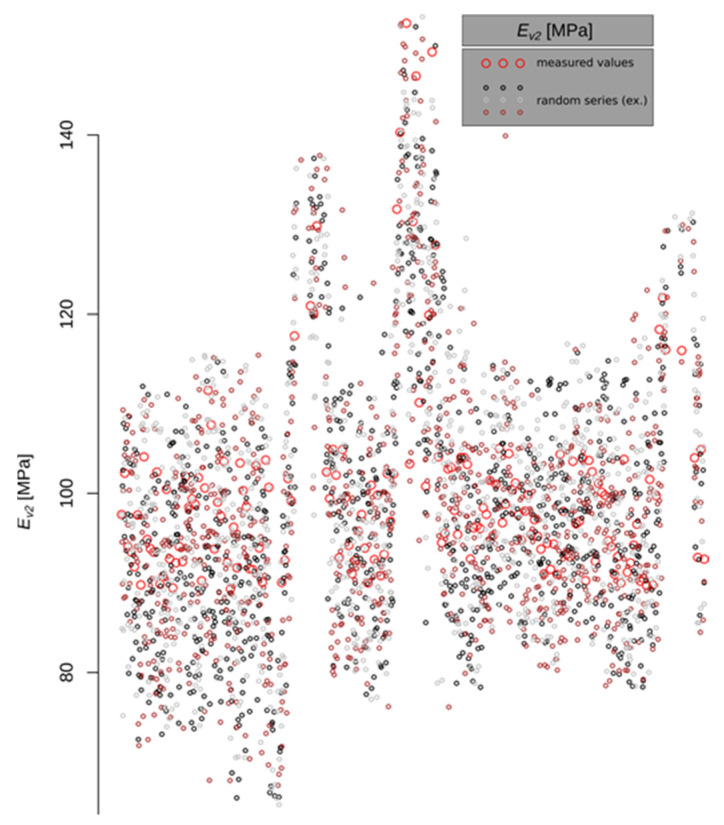
Example of the result of 3 draws of values of *E_v_*_2_.

**Figure 8:**
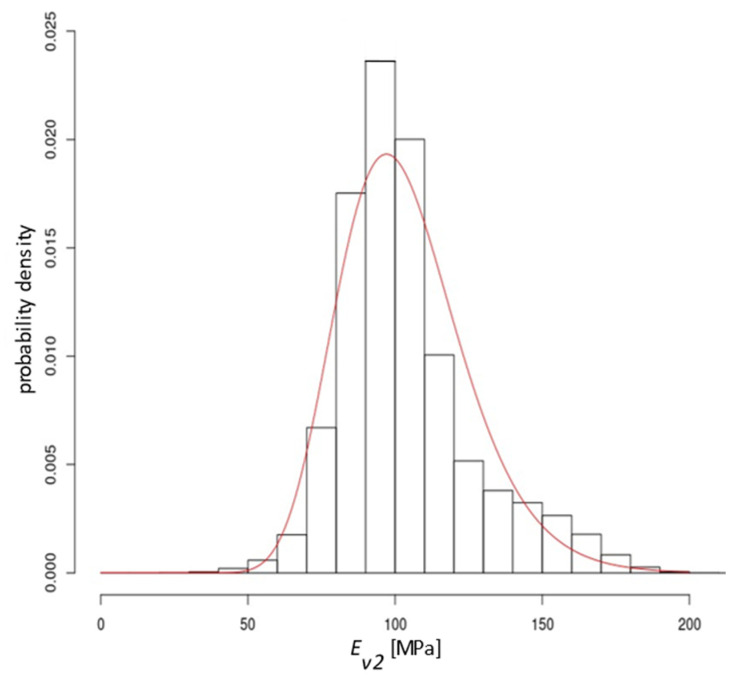
Histogram from a set of *E_v_*_2_ draw results together with the fitted log-normal probability density distribution.

The results of the sample draws are shown in Figure 7. The red circles correspond to the measured values. The black, brown and grey points represent successive realisations of the random process conditioned by the measured values of *E_v_*_2_. For the studied section 10^7^ draws were executed. Due to the very large number of points for the set of stiffness distributions, the results are presented as a histogram (Figure 8). The statistical description is presented in Table 7. A log-normal probability distribution with parameters *m* = 4.61899 and *s* = 0.2080448 was fitted to the histogram using the maximum-likelihood method. The log-normal cumulative distribution function with the determined parameters is the basis for further calculations of the failure probability.

### 3.3. Substructure Quality Assessment

**Figure 9:**
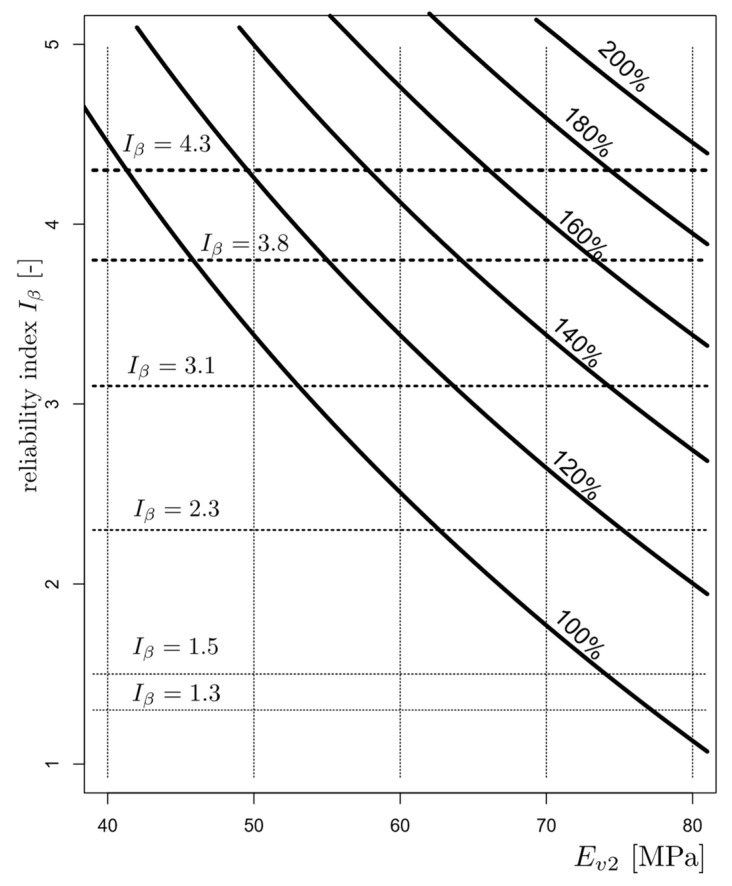
The value of the reliability index *I_β_* as a function of the expected value of the modulus *E_lim_* with respect to the level of improvement; curve 100%—present subgrade; 120%; 140%; 160%; 180%; and 200% of *E_v_*_2_.

For the railroad under investigation, a quality assessment was carried out prior to the planned modernisation according to the procedure outlined above. With the assumed value of *E_lim_*, the reliability index *I_β_* of the substructure was determined directly from Equation (5). The results are shown in Figure 9, where the dependence of the reliability index on the adopted boundary condition *E*_2*lim*_ is indicated. Horizontal lines represent the safety levels of the reliability index. This figure also illustrates the effect of the technique of uniform improvement of the whole section on the value of the reliability index. The following lines correspond to curves for levels of subgrade improvements from 120% to 200% of the initial value, respectively. Assuming an *E_lim_* value of 60 Mpa, the reliability index of the existing subgrade is 2.52. For a planned K0-class line, this value is insufficient. In order to obtain an index value of 3.1, the stiffness of the subsoil must be increased proportionally by 15% of its initial value. An index of 3.8 requires the stiffness of the subgrade to be increased proportionally by 30% and an index of 4.3 requires a 40% increase. If a boundary modulus of 70 MPa is required, the reliability index of the existing substrate decreases to 1.80. In order to obtain reliability index values of 3.1, 3.8 and 4.3, the stiffness of the substrate must be increased proportionally by 30%, 50% and 70%. However, the strategy of strengthening the whole section is rarely applied and not very effective. In practice, the methods of improving selected fragments of the route section are more frequent.

**Figure 10:**
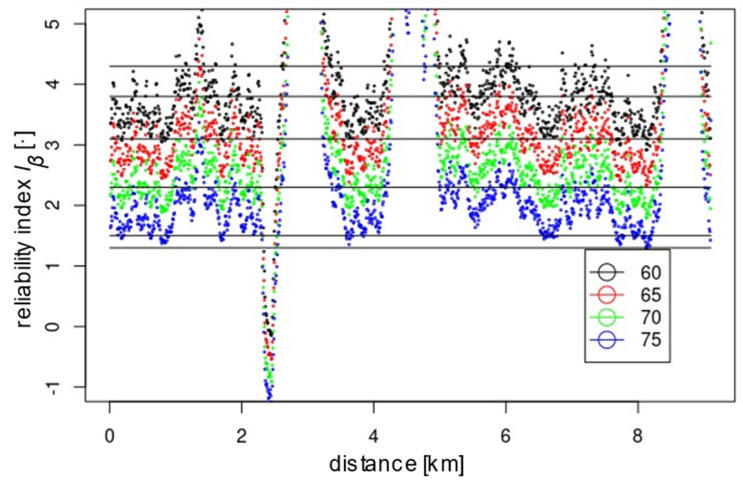
The calculated values of the reliability index *I_β_* for the full length of the investigated line for the modulus *E_lim_* = 60, 65, 70 and 75 MPa.

**Figure 11:**
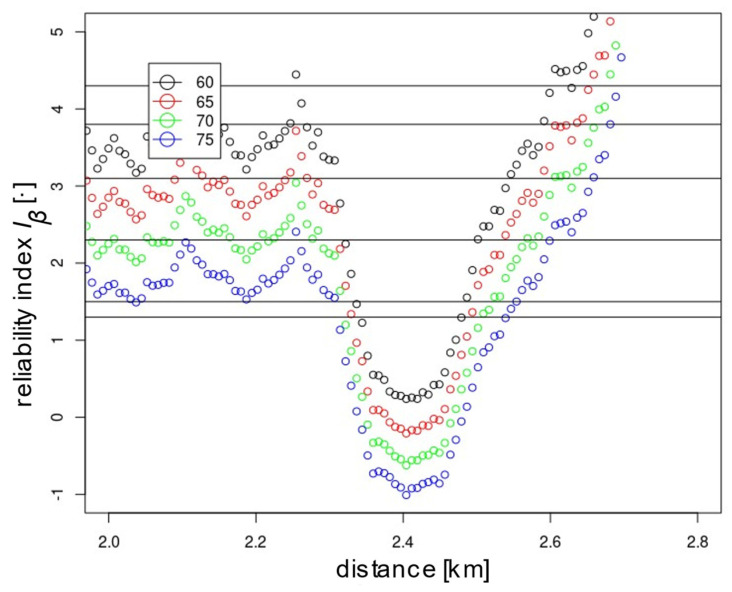
The calculated values of the reliability index *I_β_* for the 2.0–2.8 km section of the investigated line for the modulus *E_lim_* = 60, 65, 70 and 75 MPa.

In addition to meeting the global reliability condition, it is also necessary to meet it locally. For the assumed upgraded line, point-by-point reliability analyses were carried out for the assumed 7.5 m section spacing with different *E_lim_* values, i.e., 60, 65, 70 and 75 Mpa. Figure 10 presents the calculated reliability index values for the whole line section, i.e., 0–9100 m. On this basis, sections requiring reinforcement may be identified. In this case, a significant weakening of the substrate was found at 2000–2800 m, which is shown in Figure 11. Maintaining the line at K1 level with a required *I_β_* of 2.3 and a boundary modulus of 60 MPa requires additional improvement works on section 2350–2500 despite the global reliability index of the line being 2.52. To upgrade the line to K0 with an *I_β_* of 3.1, improvement is required on section 2300–2550. After the modification of the section indicated, the reliability of the examined section should be reassessed. The presented methodology can significantly influence the economics and rationality of the subgrade improvement, i.e., reduce costs and implementation time by limiting works to selected sections that do not meet the adopted reliability criterion. For the assumed value of *E_lim_* and *I_β_*, modification of the subsoil is required for a certain section. In the case of a deterministic approach, improvement is necessary for all sections where the required value was not obtained. The method also enables numerical simulations of the expected results of the improvement depending on the applied approach, i.e., proportional increase in stiffness for the whole examined section or improvement of only the fragments indicated in the condition discrepancy report. The prognosis may already be performed at the research stage, guaranteeing the appropriate level of safety of the structure, adjusted to the tasks assigned or the costs incurred for its execution. Depending on the analysis results obtained, the appropriate improvement technology should be selected. It is worth noting that the value of the reliability index *I_β_* cannot be verified by field experiments. In order to validate the method, verification should be carried out on the values of the deformation modulus obtained from the probabilistic method.

By analysing the location of the points representing the values of the deformation modules in Figure 3 and Figure 7 and the reliability index in Figure 10, it can be seen that they have a similar distribution. This characteristic dependence results from the applied geostatistical method. The drawn values of the deformation modulus (Figure 7) are autocorrelated with the experimental values obtained by the static plate load test (Figure 3). It is especially visible in places where local extremes occur. Due to this, the values obtained by drawing are a very reliable reflection of the actual values of the deformation modules. Figure 10 shows the values of the reliability index *I_β_*, which were calculated on the basis of data from Figure 7, hence the similarity of the distribution of points between these figures.

## 4. Conclusions

The paper presents the concept of objective and effective assessment of the condition of the railway track substructure with an example of application to a real example of a route under modernisation.

In the study, the railway track was treated as a reliability system based on the summation of probabilities of occurrence of modes. According to the results of static load plate tests, a spatial variation model is applied, with the use of semivariograms, to describe the ground stiffness dependencies. The possible values of the moduli between experimental points were described using a Gaussian random field conditioned by variogram. Calculations were performed using the Crude Monte Carlo method. This led to the determination of the reliability index of the substructure. In the example of the modernised railway line, the results of the applied method are presented for the given conditions (boundary model of the substructure *E_v_*_2_ and reliability index *I_β_*). The analysis was carried out with a view to both an overall uniform improvement of the line substructure and a search for problematic sections. The existence of a section that requires improvement was identified. Results are presented in relation to the input parameters adopted. In the example, the change in the extent of works does not differ significantly from the deterministic approach due to the choice of section. In the case of a more complex line structure, the results would be more conclusive, but the purpose of the paper was to show the algorithm’s functioning and to determine the necessary improvement conditions and their influence on the quality of the section.

An important distinguishing feature of the method is the estimated value of the reliability index, which unifies the design and construction process in accordance with standards. The scale based on the reliability index is compliant with the standard provisions of the Eurocode and at the same time can be scaled to the limit values of physical quantities defined in national standards. The reliability-based design has been implemented in many areas of geotechnical engineering, but in rail transport the process is progressing unevenly and is not yet strongly supported by standards documents and industry recommendation. The postulated safety levels for railway lines are the beginning of the discussion and classification. It is proposed that they should be selected or modified to correspond to regional (national) cost and risk structures. Setting them at a uniformly high level may block the development of railways, especially in less-developed countries.

The reliability approach provides clear criteria for determining the quality of railway subgrade. The presented method allows one to reduce the number of measurements, speed up the control process, determine the required scope of repair works and support the selection of the most effective improvement methods through successive simulations of possible scenarios.

An additional advantage of the approach is the use of open tools for building geostatistical models and random sampling without licence restrictions (R, Random Fields, GStat). However, in the case of very high variability of the substrate, this method may give inaccurate results and require additional tests. The presented concept is operating only on the serviceability limit-state function. The introduction of the subgrade–rail interaction, in which it will be possible to analyse ultimate limit states, is a desired direction of further development of the technique.

## Figures and Tables

**Table 1:** Theoretical models for the semivariogram.

Theoretical Model	Semivariogram	
nugget	$\gamma \left(h\right)=\left\{\begin{array}{c} 0\\ s \end{array}\right.$	when *h* = 0 when *h* > 0
linear with sill	$\gamma \left(h\right)=\left\{\begin{array}{c} \frac{sh}{r}\\ s \end{array}\right.$	when *h* ≤ *r* when *h* > *r*
spherical	$\gamma \left(h\right)=\left\{\begin{array}{c} s\left[1.5\frac{h}{r}-\kappa {\left(\frac{h}{r}\right)}^{3}\right]\\ s\;\;\;\;\;\;\;\;\;\;\;\;\;\;\;\;\;\;\;\;\;\;\;\;\;\;\;\;\;\; \end{array}\right.$	when *h* ≤ *r*
		when *h* > *r*
exponential	$\gamma \left(h\right)=s\left(1-e^{\frac{-h}{r}}\right)$	
logarithmic	$\gamma \left(h\right)=\left\{\begin{array}{c} \frac{sh}{r}\\ s \end{array}\right.$	when *h* = 0 when *h* > 0

κ—model constant typical equal 0.5 [∙].

**Table 2:** Reliability index target value for the lifetime of an object [22].

Relative Cost of Safety Measures	Failure Consequences			
	Small	Some	Moderate	Great
high	0.0	1.5	2.3	3.1
moderate	1.3	2.3	3.1	3.8
low	2.3	3.1	3.8	4.3

**Table 3:** Unit cost of railway lines of different types for selected terrain difficulties (including all cost components) in MEuro/km [44].

Type of Track	Easy Topography	Average Topography	Difficult Topography
single 100 km/h	1–3	3–15	15–40
double 100 km/h	1–4	3–20	20–50
double 300 km/h	2–6	6–30	30–50

**Table 4:** Proposed classification of the target reliability index for subgrade, taking into account the classification of the railway lines and the costs (without taking into account the terrain and excluding the high-speed lines).

Relative Cost of Safety Measures	Classification of Railway Lines		
	Secondary (K2) and Local (K3)	Primary (K1)	Trunk (K0)
	<3 MEuro/km	3–15 MEuro/km	>15 MEuro/km
high	1.5	2.3	3.1
moderate	2.3	3.1	3.8
low	3.1	3.8	4.3

**Table 5:** Results of the static load plate test on the selected section.

	Minimum Value	First Quartile	Median	Mean	Third Quartile	Maximum Value	Standard Deviation
*E_v_*_1_ [MPa]	28.99	54.35	59.40	62.68	66.10	125.90	14.29
*E_v_*_2_ [MPa]	48.70	93.33	98.52	104.99	103.86	196.50	22.53

**Table 6:** Theoretical semivariograms.

Value	Model	Nugget [MPa^2^]	Sill [MPa^2^]	Range [km]	Kappa [−]
*E_v_*_1_	exponential	69.403	204.720	0.529	0.5
*E_v_*_2_	exponential	75.254	510.740	0.306	0.5

**Table 7:** Statistical description of the draws of values *E_v_*_2_.

Draws Number	Mean	Standard Deviation	First Quartile	Third Quartile	Minimum Value	Maximum Value
1215000	103.6	22.37	89.26	112.01	21.98	214.28

## Data Availability

Data available on request due to restrictions, e.g., privacy or ethical.
